# Impact of organizational factors on adherence to laboratory testing protocols in adult HIV care in Lusaka, Zambia

**DOI:** 10.1186/1472-6963-12-106

**Published:** 2012-05-02

**Authors:** Sarang Deo, Stephanie M Topp, Andrew O Westfall, Matimbo M Chiko, Chibesa S Wamulume, Mary Morris, Stewart Reid

**Affiliations:** 1 Indian School of Business, Hyderabad, India; 2 Centre for Infectious Disease Research in Zambia, Lusaka, Zambia; 3 University of Alabama, Birmingham, AL, USA; 4 Lusaka District Health Management Board, Lusaka, Zambia; 5 Malaria Case Management Officer National Malaria Control Commission, Lusaka, Zambia; 6 Nossal Institute for Global Health, University of Melbourne, Melbourne, Australia; 7 University of North Carolina at Chapel Hill, Chapel Hill, NC, USA

## Abstract

**Background:**

Previous operational research studies have demonstrated the feasibility of large-scale public sector ART programs in resource-limited settings. However, organizational and structural determinants of quality of care have not been studied.

**Methods:**

We estimate multivariate regression models using data from 13 urban HIV treatment facilities in Zambia to assess the impact of structural determinants on health workers’ adherence to national guidelines for conducting laboratory tests such as CD4, hemoglobin and liver function and WHO staging during initial and follow-up visits as part of Zambian HIV care and treatment program.

**Results:**

CD4 tests were more routinely ordered during initial history and physical (IHP) than follow-up (FUP) visits (93.0 % vs. 85.5 %; p < 0.01). More physical space, higher staff turnover and greater facility experience with ART was associated with greater odds of conducting tests. Higher staff experience decreased the odds of conducting CD4 tests in FUP (OR 0.93; p < 0.05) and WHO staging in IHP visit (OR 0.90; p < 0.05) but increased the odds of conducting hemoglobin test in IHP visit (OR 1.05; p < 0.05). Higher staff burnout increased the odds of conducting CD4 test during FUP (OR 1.14; p < 0.05) but decreased the odds of conducting hemoglobin test in IHP visit (0.77; p < 0.05) and CD4 test in IHP visit (OR 0.78; p < 0.05).

**Conclusion:**

Physical space plays an important role in ensuring high quality care in resource-limited setting. In the context of protocolized care, new staff members are likely to be more diligent in following the protocol verbatim rather than relying on memory and experience thereby improving adherence. Future studies should use prospective data to confirm the findings reported here.

## Background

Operational research studies have recently demonstrated the feasibility of large scale ART programs within public sector, based on satisfactory clinical outcomes such as survival and treatment success [1-3]. While population health *outcomes* are a natural focus of health services research seeking to determine the effectiveness of a public health intervention, they are determined by multiple factors classified under “quality of process” and “quality of structure” [4]. Quality of *process* relates to the actual delivery of care, including adherence to proven standards of care (diagnostic and treatment testing algorithms and guidelines) and the ability to communicate and build trust with patients [5]. Quality of *structure* relates to physical characteristics (facilities and equipment, how they are organized and managed, the operating hours of the facility etc.) and human characteristics (skills and qualifications of the staff and their level of motivation and job satisfaction) of the organizational environment within which care is delivered. The structure-process-outcome framework [4] relates these components to final patient outcomes.

Motivated by chronic human resource shortages in rapidly growing HIV treatment programs in sub-Saharan Africa [2], health services researchers have followed two broad lines of inquiry into quality of care. First, recent studies have measured structural factors of service delivery such as worker satisfaction and motivation [6-9] and have uncovered a number of predictors of dissatisfaction. These include public vs. private sector employment, workload, availability of resources, salaries [10], and low levels of staffing, management support and control over their practice [11]. Second, other studies have documented the gap between desired and actual quality of care along structure, process or outcome dimensions [12-14]. However, few studies have examined whether structural and organizational factors are determinants of quality of care in these resource-limited settings.

Research from developed countries, focusing on surgical procedures in acute care settings, suggests that motivation and burnout levels of staff can contribute significantly to differences in quality of care [15,16]. However, specific components of that can impact the quality of care vary across different settings [17]. In developed countries, these include complexity of medical technology, number of chronic non-communicable diseases and expensive and fragmented healthcare delivery systems [5,18,19]. In developing countries, additional factors that have been suggested include weak physical infrastructure, poor professional working environment and healthcare worker shortages [17,20].

This study seeks to address a gap in the literature by exploring the relationship between structural factors and quality of process in the context of HIV care and treatment delivery in Zambia. We investigate how physical space, level of staffing, staff burnout, staff absenteeism, staff experience and facilities’ experience with ART provision are associated with levels of adherence to clinical protocol (in this case national guidelines requiring certain laboratory tests) as part of Zambian HIV care and treatment program.

### Program description: HIV care and treatment

In Lusaka, Zambia, a large-scale public sector HIV care and treatment program has been run by the Zambian Ministry of Health (MOH) since April 2004. Clinical care, patient tracking, and outcomes monitoring for the Lusaka program are standardized across all primary healthcare facilities aligned with national guidelines for adult HIV treatment [3,21]. Within each facility, ART departments are usually staffed by two to six nurses and supported by two to five lay healthcare workers. This is comparable to the number of healthcare professionals in typical maternal and child health departments, but higher than that in the outpatient departments in the same clinics. At enrollment, patients undergo physical examination to determine WHO stage of HIV infection. This, with the patient’s CD4 count and other investigations, determines the extent of immune suppression and the eligibility to start antiretroviral therapy. Other blood tests are done during follow-up visits as required by national guidelines.

In the clinics in this study, nursing staff collected all samples on-site. Samples were labelled and collected according to a twice-daily schedule, and transported to the Centre for Infectious Diseases Research in Zambia (CIDRZ) central laboratory for processing. All results were processed before being printed in hard copy and returned to clinics using the same twice-daily transport schedule between 5–10 working days. Hard copy results were then entered electronically in patients’ files by on-site data entry clerks and finally filed in patients’ medical files by registry staff. Availability of tests including commodities in Lusaka was extremely reliable, and there were no financial barriers for testing. Provision of all care and treatment in the ART clinics was free, including all laboratory investigations. However, logistical breakdowns in the labelling and transport of specimens, the return of results and their entry into the patient database should be considered as limitations in our causal analysis.

## Methods

### Study population

#### Setting

Of the more than 150 HIV care and treatment facilities run by MOH in Lusaka Urban District, we focused on 13 because of the similarity in geographic location, conditions of service-delivery for these clinics and, availability of data on staff burnout in these facilities from a healthcare worker survey [9] conducted between March and June 2007. As most of these clinics had been providing HIV care for over 6 months (one clinic only 5 months), the effects of initial scale-up were minimized. Our study was approved by the institutional review boards at Northwestern University, University of Alabama at Birmingham, and the Research Ethics Committee of the University of Zambia.

#### Patients / Visits

We included all recorded visits of adult patients (aged 16 years or more) diagnosed with HIV and enrolled in care during the calendar year 2007. This coincides with the time frame of the healthcare worker survey [9].

### Data sources

#### Staff motivation survey

Data on predictors related to staff motivation (burnout, experience, absenteeism and turnover), were obtained from the healthcare worker survey [9] in various primary health departments (maternal and child health, outpatient, inpatient, labor, HIV care and treatment, tuberculosis) in Lusaka Urban District in 2007. Approximately 500, anonymous responses from 13 facilities were received and analyzed. Some of the relevant questions are reproduced in Table 1.

**Table 1:** Description of predictor variables and their data sources

**Measure**	**Description**	**Data Source**
Staff Burnout	A continuous variable at the clinic level to measure the level of burnout on a standardized scale experienced by the staff at the clinic.	Healthcare worker survey about staff motivation and health seeking behavior (Kruse et al. [9])
	For each clinic burnout was defined as the median of the individual responses to the following question in the healthcare worker survey: “Q-32 Using your own definition of ‘burnout,’ please circle ONE of the following:	
	1. I have no symptoms of burnout.	
	2. Sometimes I am under stress, but I don’t feel burned out.	
	3. I am definitely burning out and have occasional symptoms of burnout.	
	4. The symptoms of burnout that I’m experiencing won’t go away.	
	5. I feel completely burned out. I am at the point where I need to make some changes or seek some sort of help.	
Staff experience	A continuous variable at the clinic level to measure the work experience of healthcare workers at the facility.	Healthcare worker survey about staff motivation and health seeking behavior (Kruse et al. [9])
	For each clinic experience was defined as the median of the individual responses to the following question in the healthcare worker survey:	
	“Q-7 Amount of time in your current position (write the number of months and/or years):	
	______ MONTHS ______ YEARS”	
Staff absenteeism	A continuous variable at the clinic level to measure the extent of absenteeism	Healthcare worker survey about staff motivation and health seeking behavior (Kruse et al. [9])
	Individual responses to the following 4 questions in the healthcare worker survey were summed to determine each worker’s total number of days absent. For each clinic absenteeism was defined as the median of these individual summed responses: “Q-12 Have you been away from work because of funerals”	
	“Q-13 Have you been away from work because you were ill”	
	“Q-14 Have you been away from work because a family member was ill”	
	“Q-15 How much leave (local and accumulated) have you taken in the last 12 months”	
Staff Turnover?	Q9 from the HCW		
	How many healthcare workers in your department have left their position in the last 2 years		
Clinic age	A continuous variable at the clinic level to measure the time since initiation of ART program at a particular health facility	Electronic medical record data	
Staffing ratio	A continuous variable at the clinic level for each month in calendar year 2007 to measure the number of patient visits per shift.	CIDRZ overtime payment data	
	This was calculated by dividing the total number of patient visits in a month by the total number of FTE shifts paid for in that month.	Electronic medical record data	
Physical space	A continuous variable to measure the availability of physical space.	Facility architectural plans	
	This was calculated by dividing the floor area of each facility measured in sq. meters divided by average daily visits to the facility	Electronic medical record data	

#### Electronic medical records

In addition to obtaining data on the outcomes of the study, electronic medical records (SmartCare database) were used to obtain data on the number of daily visits to facilities. The study did not require identification of patients; rather the primary record marker was the type of visit – ART initiation, ART follow up etc. Visit information was extracted based on inclusion / exclusion criteria mentioned above and all patient identifiers were removed.

#### Others

Records of overtime payments were used to calculate the number of shifts worked by nurses and clinical officers in ART clinics. In 2007, all staff members working in the ART clinics were deputed from other departments and worked overtime in the ART clinics. Administrative databases within CIDRZ and MOH were used to calculate the length of time since initiation of ART program services at each facility. Architectural plans of each facility were used to calculate the total floor area of each clinic, to determine calculation of physical space.

### Measures

#### Outcomes

Owing to shortages of physicians, clinical officers (analogous to physician’s assistants in the U.S.) and nurses delivered majority of healthcare services in our setting. To ensure a minimum standard of care, clinicians followed visit-specific protocols (initial visit form, routine follow-up form etc.) that reflected national treatment guidelines. These forms were designed to guide clinicians through initial evaluation of newly enrolling patients and all subsequent follow-up visits. In this study, we constructed dichotomous variables to indicate whether appropriate laboratory / clinical tests were conducted at each of these visits.

**Initial History and Physical (IHP) visit:** At the initial visit, we examined whether patients were correctly assessed according to WHO and national guidelines. In addition we examined whether baseline laboratory investigations, such as CD4, hemoglobin, liver function tests were properly carried out in accordance with WHO staging and national guidelines. We gave a positive credit to the facility if results were recorded in the patient’s chart within 4 weeks before and after the patient’s visit.

**Follow-up (FUP) visits:** We chose CD4 test as a measure of adherence to follow-up visit protocol because CD4 count is a key clinical indicator of treatment response (for those on treatment) and disease progression (for those not on treatment). Thus, it provides a better measure of adherence to protocol across all patients compared to other non-compulsory tests such as hemoglobin and liver function [22]. We measured whether repeat CD4 testing was ordered within 6 months of the previous test. To account for variability in patient attendance to scheduled visits, we developed the following rule. For each follow-up visit, we *expected* a CD4 count test to be done if there was no CD4 result entered in the database in the preceding 160 days. For each visit where CD4 was expected, we considered the CD4 test *done* if the result was recorded in the patient’s chart within four weeks after the visit. Our quality measure was calculated as the total CD4 tests *done* in each month, divided by the sum of visits where a CD4 was *expected* and *not expected but done* in each month for each facility. For sensitivity analysis, we repeated the analysis with 180 days time window. We also repeated the analysis with a different definition of *done* as either a tick mark on the patient’s chart or a result within 4 weeks. In addition, we also counted the CD4 test as *done* in follow-up visits where it was *not expected* according to our definition above.

We did not have access to the identity of health care workers involved in provision of care to individual patients. Thus, we could not analyze the difference in adherence to protocols at the worker level but could only infer these differences at the facility level.

#### Predictors

Facility level measures for staff burnout, staff experience, staff absenteeism and staff turnover were calculated by taking the median of individual responses to the staff motivation survey from that facility. Monthly staffing ratio was calculated by dividing the patient visits to the ART department by full time equivalent (FTE) staff shifts. This included nurses, physicians, clinical officers, technicians and pharmacists. A measure of physical space was calculated by dividing the floor area of ART department by the total patient visits during 2007. An alternative measure using average patient visits per day for each month did not alter the results substantially. In our setting, more than 90 % of the space was used for delivery of care and the rest for administrative tasks. Clinic age was calculated as the time since the initiation of ART program services as of January 2007 (Table 2). All predictors, except those constructed from health worker survey, pertained to ART services. The predictors derived from the survey included staff members belonging to other departments as well.

**Table 2:** Summary statistics of predictor variables

**Site**	**Staff experience (years)**	**Staff turnover (number within past two years)**	**Staff absenteeism (days per staff in past 12 months)**	**Staff burnout (scale of 1–5)**	**ART Clinic age (months)**	**Physical space (m**^**2**^**per average number of visits per day)**	**Number of patient visits per month per FTE shift Mean (SD)**
Bauleni	12.7	3.0	16.0	3.1	24.4	1.6	4.3 (0.4)
Chawama	10.7	3.0	15.0	2.7	10.1	3.0	6.9 (1.4)
Chelstone	12.0	3.0	18.0	2.6	32.1	0.4	11.0 (1.2)
Chilenje	6.8	2.0	16.5	2.8	28.0	0.5	7.1 (0.9)
Chipata	9.5	2.0	6.0	3.6	23.0	7.6	12.7 (1.6)
George	11.0	3.0	17.0	3.3	29.0	3.0	8.0 (0.9)
Kabwata	7.3	2.0	15.0	2.9	9.5	7.8	4.0 (0.2)
Kalingalinga	5.0	2.5	18.0	2.8	32.1	2.6	10.2 (1.6)
Kamwala	4.9	3.0	15.0	2.9	29.0	3.3	10.5 (1.1)
Kanyama	9.9	2.0	17.0	3.0	32.1	0.9	9.5 (0.8)
Matero Main	6.9	3.0	17.5	3.2	5.0	5.0	3.8 (0.5)
Matero Reference	9.8	2.0	16.0	2.6	29.0	1.4	10.5 (1.5)
M’tendere	13.0	2.0	14.0	3.2	32.1	0.6	7.8 (0.7)

Each column shows the median value for each variable by site. Measures of these predictor variables were held constant for each site during the study period.

### Statistical analysis

We ran multi-level logistic regression models using SAS GLIMMIX procedure for visit level outcome variables. We used facility-month combinations to define the hierarchical structure, intercept as a random effect, and other predictors as fixed effects. We developed and analyzed two model variants (nested within each other) to assess the incremental impact of different predictor variables: (i) Model 1 included Staff Experience, Staff Turnover, Space per visit, Clinic Age, Visits per shift, (ii) Model 2 included all the above predictors and Staff Absenteeism and Staff Burnout. We did not include burnout in the first model since it was assessed using a single item from the healthcare worker survey, whose validation with a more accepted Maslach Burnout Inventory has been conducted outside of resource-limited setting [23]. Similarly, we did not include absenteeism in the first model since it itself can be considered as an outcome of other staff related variables and its direct impact on health outcomes was, *a priori,* not clear. Since the results were not very different for the two model variants and because the coefficients of absenteeism and burnout were significant, we present the results of Model 2. All analyses performed using SAS/STAT software, Version 9.1 (Cary, NC, USA).

## Results

### Summary statistics for predictors

Facilities varied considerably in the median experience of their staff, ranging from 4.9 years to 13 years. There was less variation in other staff-related measures calculated from the healthcare worker survey such as staff burnout, staff turnover and staff absenteeism. Facilities’ experience with HAART ranged from 5 months to 32.1 months as of January 2007. Physical space (from 0.4 m^2^ per visit per day to 7.8 m^2^ per visit per day) and staffing ratios (from 3.8 to 12.7 patient visits per FTE shift) varied substantially across facilities (Table 2). In addition, there was considerable variation over time within facilities (Additional file 1).

### Outcomes for initial visits

During 2007, there were 20,441 IHP patient visits (Table 3). Of these, CD4 and HB test results were available within 4 weeks for 85.4 % and 84.2 % of the visits respectively. WHO disease staging was performed in 99 % of the visits and a liver function test was done for 80.7 % of the patients with WHO stage IV disease. There was considerable variation among different study facilities for all IHP outcomes, except WHO staging. The minimum for each outcome was as follows: 79.5 % for CD4, 77.1 % for HB and 96.47 % for WHO staging. Hierarchical logistic regression showed that the odds of conducting CD4 test decreased with staff absenteeism, staff burnout and staffing ratio (visits per FTE shift) while the odds increased with turnover, physical space, and clinic age. The odds of conducting HB tests decreased with staff burnout, and staffing ratio (visits per FTE shift) while the odds increased with staff experience, staff turnover, physical space and clinic age (Table 4).

**Table 3:** Summary statistics for outcomes during initial health and physical (IHP) visit

**Site of IHP**	**Number of adult IHP’s in 2007**	**Percent of IHP’s with CD4 count ordered or result recorded within +/− 4 weeks**	**Percent of IHP’s with Hemoglobin count ordered or result recorded within +/− 4 weeks**	**Percent of IHP’s with WHO Stage done**	**Number of WHO Stage IV IHP’s in 2007**	**Percentage of Stage IV IHP’s with Liver Function Test ordered or result recorded within +/− 4 weeks**
Bauleni	521	88.68 %	89.44 %	97.9 %	25	84.00 %
Chawama	2,352	87.71 %	88.05 %	98.8 %	129	86.05 %
Chelstone	1,614	85.19 %	85.25 %	96.5 %	44	79.55 %
Chilenje	1,356	83.92 %	85.03 %	99.6 %	78	85.90 %
Chipata	1,980	86.31 %	80.05 %	99.4 %	218	80.28 %
George	1,816	90.25 %	90.47 %	99.3 %	112	77.68 %
Kabwata	787	89.33 %	89.33 %	99.5 %	24	75.00 %
Kalingalinga	934	84.05 %	77.09 %	98.1 %	43	76.74 %
Kamwala	1,788	89.82 %	83.33 %	99.4 %	62	72.58 %
Kanyama	2,566	79.54 %	78.88 %	99.2 %	144	75.69 %
Matero Main	1,342	85.77 %	86.51 %	99.4 %	89	86.52 %
Matero Reference	2,153	82.30 %	83.14 %	98.7 %	241	82.16 %
Mtendere	1,232	82.79 %	82.87 %	98.5 %	66	80.30 %
Total	20,441	85.43 %	84.16 %	98.9 %	1,275	80.71 %

**Table 4:** Multi-variable model for predictors of not ordering a test

	**Follow-up visit (FUP)**	**Initial health and physical visit (IHP)**			
**Label**	**CD4 count**	**CD4 count**	**Hemoglobin**	**WHO staging**	**Liver function**
Staff experience	0.93^***^ (0.92, 0.96)	1.00 (0.96, 1.04)	1.05^***^ (1.01, 1.09)	0.90^**^ (0.81, 1.00)	1.00 (0.91, 1.09)
Staff turnover	1.45^***^ (1.25, 1.67)	1.69^***^ (1.39, 2.08)	1.43^***^ (1.18, 1.72)	0.78 (0.47, 1.30)	0.94 (0.59, 1.52)
Staff absenteeism	0.97^*^ (0.93, 1.00)	0.94^**^ (0.89, 0.99)	0.98 (0.93, 1.03)	0.93 (0.82, 1.08)	0.96 (0.85, 1.08)
Staff burnout	1.14^**^ (1.00, 1.30)	0.78^***^ (0.64, 0.94)	0.77^***^ (0.64, 0.93)	0.88 (0.51, 1.54)	1.06 (0.65, 1.75)
Physical space	1.03 (0.98, 1.09)	1.12^***^ (1.04, 1.20)	1.10^***^ (1.02, 1.19)	1.08 (0.85, 1.35)	0.87 (0.71, 1.06)
Clinic age	1.41^***^ (1.20, 1.64)	1.43^***^ (1.15, 1.79)	1.23^**^ (1.00, 1.54)	1.05 (0.59, 1.89)	0.64^*^ (0.38, 1.06)
Staffing ratio	0.93^***^ (0.90, 0.95)	0.93^**^ (0.89, 0.97)	0.93^***^ (0.88, 0.96)	0.93 (0.83, 1.04)	1.00 (0.90, 1.10)

Values indicate odds of not ordering a test per unit increase in the independent variable) Parentheses contain the 95 % confidence interval. ^***^ indicates that the coefficient is significant at 1 % level, ^**^ indicates that the coefficient is significant at 5 % level and ^*^ indicates that the coefficient is significant at 10 % level.

### Outcomes for follow-up visits

There were 136,432 follow-up visits in our study facilities during 2007. A CD4 test was *expected* in 34,150 follow-up visits according to the rule described earlier, of which, a test was *done* in 21,323 visits (62.44 %). We excluded from our analysis 70,793 follow-up visits where the test was *not expected* and *not done and* 31,489 follow-up visits, where the test was *not expected* but was *done.* In all facilities, this was significantly lower than the percentage of CD4 tests conducted in IHP visits (Table 5).

**Table 5:** Summary statistics of CD4 tests during follow up visits

**Site**	**Number of adult FUP’s in 2007 (excluding visits where CD4 count not expected and not done)**	**Percent of FUP’s with CD4 count tickbox marked or CD4 count result within +/− 4 weeks**
BAULENI^*^	635	75.28 %
CHAWAMA^*^	3,598	55.00 %
CHELSTONE^*^	3,224	61.69 %
CHILENJE^*^	1,996	71.14 %
CHIPATA^*^	3,177	66.70 %
GEORGE^*^	3,522	70.33 %
KABWATA^*^	1,585	63.79 %
KALINGALINGA^*^	2,368	70.99 %
KAMWALA^*^	2,561	69.74 %
KANYAMA^*^	3,383	53.03 %
MATERO MAIN^*^	1,226	67.78 %
MATERO REFERENCE^*^	4,247	52.93 %
MTENDERE^*^	2,628	57.46 %
TOTAL	65,639	85.5 %

* indicates that the difference is significant at 1 % level compared to the percent of IHP’s with CD4 count ordered or result recorded within +/− 4 weeks shown in Table 3. *Expected* is based on our definition, i.e., if a CD4 result was not recorded in the database up to 160 days prior to the current visit.

In the hierarchical logistic regression models, (Table 4), the odds of conducting the CD4 test in follow up visits decreased with staff experience, absenteeism and staffing ratio (visits per FTE shift) while the odds increased with staff turnover, clinic age and staff burnout. The coefficients were fairly stable across different definitions of time windows (180 days vs. 160 days), tests done (results only vs. results and tick mark vs. results or tick mark) and eligible visits (CD4 expected only or CD4 expected and not expected but conducted). We do not report them for brevity.

## Discussion

This, to our knowledge, is the first study regarding the association of structural and organizational factors with quality of HIV care in resource-limited settings. Specifically, we investigate the drivers for variation in adherence to laboratory testing protocol by health care workers across facilities. Adherence to these tests is critical to ensure good patient outcomes because WHO staging and CD4 count at initial visit are critical inputs to initiation of ART. In the presence of resource constraints, adherence to protocol and making right decisions also has implications at the population level. For instance, initiating an ineligible patient on ART without conducting all the tests also implies not initiating another eligible patient on ART. Similarly, not conducting certain tests for monitoring can have undesired consequences, especially if the frequency of these tests is low.

Our results indicate that health workers adhere to similar aspects of treatment protocol more strictly during initial visit than follow-up visits. This could be because the initial visit is more standardized whereas follow-up visits are more customized depending on the patient’s health status. Also, initial assessment can be construed as more critical since it determines treatment eligibility. Moreover, there are significant differences in the associations of organizational factors with adherence to protocol in initial versus follow-up visits.

The importance of physical space in ensuring quality of care in resource-limited settings has been mentioned before in the literature [20] and overcrowding has been cited as one of the primary drivers of inadequate care in emergency rooms [24,25]. However, ours is the first study that provides empirical support for this notion. The procedure of taking blood requires time, space and privacy, which might explain why increased space was associated with higher odds of ordering and conducting repeat CD4 test. In addition, limited physical space creates a situation of crowding, convoluted patient flow, which can aggravate and confuse staff and patients leading to compromised quality of care.

Some seemingly counterintuitive findings could be related to the design of clinical protocols with some staff cadres permitted to perform specific activities but not others. For instance, nurses are required to double-check the ordering of blood tests during FUP visits to minimize the impact of clinician oversight. This potentially explains the findings that burnout *increases* the odds of conducting CD4 test during FUP visits. It is plausible that burnout amongst clinicians, who tend to be the most overloaded and therefore most stressed, may be (over) compensated for by less burnt out nursing staff [11].

Similarly, the result that greater staff experience *decreased* and higher staff turnover *increased* the odds of conducting tests appears counterintuitive from a developed country perspective. However, we believe that this is plausible in a high workload setting with protocolized care. For example, less experienced staff members tend to adhere better to protocols and newly introduced staff may want to demonstrate their performance to supervisors by being more compliant with protocols. This effect may wane over time as workers become complacent with attention to protocol details. More experienced staff and those who have worked at a clinic for more time *may* therefore put less emphasis on following protocol, relying more on personal experience or clinical judgment. This study suggests that turnover might be beneficial if it facilitates the replacement of demotivated and burnt out staff at the facility.

The results also demonstrate that some explanatory variables influence different outcomes differently. Due to the highly protocolized nature of ART and the step-by-step nature of healthcare delivery in this setting, responsibility for certain tasks often lies with different personnel in different parts of the clinic. One of the results of this style of care is that responsibility for certain tasks is atomized, making these ‘outcomes’ susceptible to different variables. For example, some outcomes are highly dependent on the staff member responsible for registering /enrolling clients, and that staff member is often different (including potentially having different training) to those responsible for WHO staging, or for those responsible for drawing blood to follow-up on test orders.

Another plausible reason for these different effects is that faced with time constraints, the staff members might prioritize their cognitive efforts on some tasks over others based on what they believe is more important. For instance, CD4 count at the time of enrolment is considered very important for all future treatment decisions. As a result, despite heavy workload and high levels of burnout, the staff members tend to not overlook this test.

Based on the authors’ programmatic experience, noncompliance to protocols is likely to be more common amongst Clinical Officers (COs) than nurses, due to the acute bottlenecks experienced at the point of patient screening, which promoted a culture of ‘clearing’ patients as quickly as possible. Nonetheless, nurses and COs working in ART maybe more compliant to protocols compared to other departments due to more a rigorous and continuous system of quality assurance checks.

The strengths of this study include the availability of electronic clinical and laboratory data on a large numbers of patient visits. We also had access to architectural data on physical space and information on levels of health care worker burnout, which was conducted in this setting.

However, there are limitations arising from the fact that the data were not originally collected for this study. The limited scope of the study prevented us from collecting data on additional measures pertinent to our objective. One such measure is leadership of nurse in-charge. Programmatic experience of authors in Zambia strongly suggests that facility-level leadership often plays an important role in adherence to protocolized care in weak health systems. However, creating a leadership index would require conducting a survey among nurses, which was beyond our scope. Future studies should develop and/or refine existing methodologies and collect prospective data to investigate this link.

Our measure of staffing was derived from administrative (payroll) database. This almost certainly results in some overestimation of the actual level of staffing in the clinic, due to unannounced absences from the facility and the practice of getting paid for the shifts but not being physically present in the clinic. Anecdotal evidence indicates that both practices were widely prevalent among clinic staff during the study period. Our measure of absenteeism was self-reported, based on the 2007 survey of healthcare workers and it did not account for unplanned absences. Our measure of experience (number of years in a particular grade) also partly captures the effect of age of the health care worker, particularly since the ART program started only 3–4 years before the survey. Thus, our results could be interpreted to imply that younger workers are more likely to adhere to the protocol because either they are open to clinic management ideas or because their education is structurally different than their old counterparts or because they are less tied to conventional ways of doing things.

There was limited variation on some of our predictor variables such as absenteeism, burnout and turnover, which may have limited the statistical power to detect significant effects. Similarly, almost all facilities performed very well on WHO staging with very little variation across facilities. These factors might explain the lack of statistically significant results for many associations. Also, the number of patients in WHO stage IV disease was very low across all facilities, potentially explaining the lack of significant associations for laboratory investigations done.

Without access to data on outcomes at the level of individual health care workers, we are unable to comment on what factors differentiate some workers from others in being more or less adherent to protocols. Moreover, we note that the results cannot be used to assess the absolute quality of care provided at the study facilities. This is because of the lack of external benchmark on what constitutes good quality care in these resource-limited settings and because the study was not designed to answer this question. Because of imperfect patient adherence to visits, we had to allow sufficient time buffer (+/− 4 weeks) in constructing our quality measures.

Our choice of urban facilities in Lusaka, Zambia’s capital, limits the generalizability of our results in other locations, especially rural regions of the country. However, these results suggest similar assessments in other resource-limited settings attempting rapid scale-up of HIV care and treatment are necessary (particularly in sub-Saharan Africa) to ensure that the relationship between structure, process and outcomes in settings utilizing protocolized healthcare delivery are understood adequately by policy makers, donors and implementers.

## Conclusions

The results reported here challenge some established relationships between some organizational factors such as workload, burnout and experience, and the delivery of appropriate care. Further research, using prospective data, is needed to confirm these findings and understand the degree of influence that various organizational factors (structure and process) may have on the quality of care (outcomes) being delivered in high-volume, protocolized healthcare settings, such as those in many developing countries.

## Competing interests

The authors declare that they have no competing interest.

## Authors’ contributions

SD conceived the study, designed the analysis plan, interpreted the data, and wrote the manuscript. AOW provided data management and conducted statistical analyses. ST, AOW, MMC, CSW, MM, SR contributed to the data interpretation and provided critical revisions of the manuscript for intellectual content. All authors approved the final version for submission.

## Pre-publication history

The pre-publication history for this paper can be accessed here:

http://www.biomedcentral.com/1472-6963/12/106/prepub

## Supplementary Material

Additional file 1 Variation of staffing ratios in study facilities during calendar year 2007. Click here for file
