# Supplementary material for: Impact of organizational factors on adherence to laboratory testing protocols in adult HIV care in Lusaka, Zambia
Source: BMC Health Serv Res. 2012 May 2;12:106. doi: 10.1186/1472-6963-12-106 (PMC3478975; doi:10.1186/1472-6963-12-106)
Supplement: Additional file 1 — Variation of staffing ratios in study facilities during calendar year 2007. [file 1472-6963-12-106-S1.doc]

## Appendix 1: Variation of staffing ratios in study facilities during calendar year 2007

| Site | Jan 2007 | Feb 2007 | Mar 2007 | Apr 2007 | May 2007 | Jun 2007 | Jul 2007 | Aug 2007 | Sept 2007 | Oct 2007 | Nov 2007 | Dec 2007 | Mean | SD |
| --- | --- | --- | --- | --- | --- | --- | --- | --- | --- | --- | --- | --- | --- | --- |
| Bauleni | 4.6 | 4.2 | 4.1 | 3.7 | 4.2 | 4.4 | 4.5 | 4.5 | 4.0 | 5.0 | 3.8 | 4.8 | 4.3 | 0.4 |
| Chawama | 7.9 | 7.3 | 6.4 | 6.3 | 7.9 | 7.1 | 6.3 | 7.3 | 5.5 | 5.7 | 5.0 | 10.1 | 6.9 | 1.4 |
| Chelstone | 13.9 | 12.1 | 10.7 | 9.3 | 11.6 | 10.8 | 11.3 | 10.4 | 9.7 | 11.1 | 9.9 | 11.2 | 11.0 | 1.2 |
| Chilenje | 9.6 | 7.5 | 6.4 | 6.3 | 6.9 | 6.6 | 7.3 | 6.9 | 6.7 | 6.6 | 7.8 | 7.0 | 7.1 | 0.9 |
| Chipata | 14.4 | 11.0 | 10.8 | 10.6 | 15.3 | 12.3 | 14.4 | 13.1 | 11.7 | 14.2 | 12.6 | 11.9 | 12.7 | 1.6 |
| George | 8.7 | 7.0 | 7.5 | 7.4 | 7.4 | 8.7 | 6.5 | 8.0 | 7.8 | 8.2 | 8.5 | 9.9 | 8.0 | 0.9 |
| Kabwata | 4.2 | 3.9 | 3.9 | 4.0 | 4.1 | 3.9 | 4.1 | 4.4 | 3.7 | 4.0 | 3.8 | 3.9 | 4.0 | 0.2 |
| Kalingalinga | 10.6 | 11.7 | 9.9 | 9.5 | 12.7 | 8.8 | 10.1 | 12.2 | 11.3 | 9.8 | 7.5 | 7.9 | 10.2 | 1.6 |
| Kamwala | 11.2 | 9.1 | 12.5 | 10.8 | 11.7 | 11.1 | 10.2 | 10.6 | 9.3 | 9.6 | 9.5 | 9.9 | 10.5 | 1.1 |
| Kanyama | 10.3 | 9.5 | 9.2 | 10.7 | 9.1 | 9.4 | 10.6 | 10.3 | 8.5 | 8.8 | 8.7 | 8.8 | 9.5 | 0.8 |
| Matero Main | 3.6 | 3.3 | 3.4 | 3.2 | 4.1 | 3.5 | 3.4 | 3.7 | 3.6 | 3.8 | 4.0 | 5.2 | 3.8 | 0.5 |
| Matero Reference | 11.0 | 10.8 | 10.7 | 9.6 | 12.5 | 13.0 | 11.3 | 11.9 | 8.6 | 9.4 | 8.3 | 9.0 | 10.5 | 1.5 |
| Mtendere | 7.6 | 7.8 | 7.6 | 7.2 | 7.9 | 9.2 | 8.1 | 8.3 | 8.2 | 7.2 | 6.6 | 8.0 | 7.8 | 0.7 |
